# Designing Advanced Cross-Linked Proton Exchange Membranes with Enhanced Structural Homogeneity and Proton Conductivity via Radiation-Induced RAFT Polymerization

**DOI:** 10.1021/acsomega.4c01522

**Published:** 2024-06-18

**Authors:** Feyza Genç, Nazlıcan Yıldırım Kılıç, Murat Barsbay

**Affiliations:** † Polymer Chemistry Division, Department of Chemistry, Faculty of Science, Hacettepe University, 06800 Ankara, Turkey; ‡ Polymer Science and Technology Division, Institute of Science, Hacettepe University, 06800 Ankara, Turkey

## Abstract

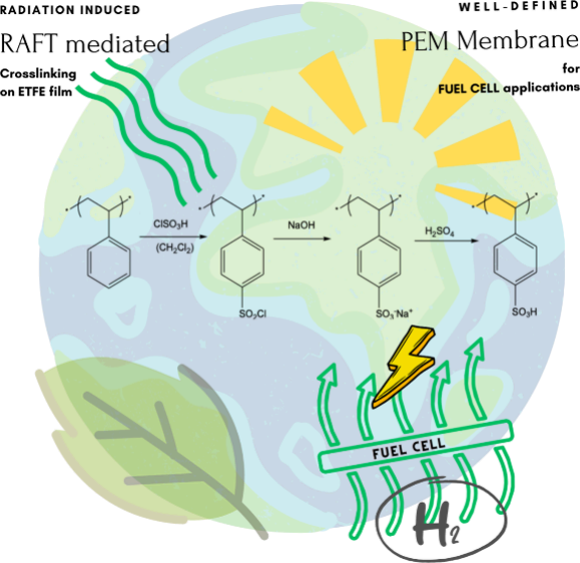

This study introduces an innovative approach to fabricate well-defined cross-linked proton exchange membranes (PEMs) using radiation-induced reversible addition–fragmentation chain transfer (RAFT)-mediated polymerization on cost-effective ethylene tetrafluoroethylene (ETFE) films. The incorporation of the RAFT mechanism into the cross-linking process significantly enhanced structural homogeneity, providing uninterrupted proton conductivity. Thorough characterizations confirmed the successful grafting of polystyrene (PS) chains onto ETFE films and subsequent sulfonation. Despite a reduction in proton conductivity attributed to restricted chain movements, a notable improvement in chemical stability was observed after cross-linking reactions. Chemical stability of the cross-linked membranes increased approximately 4-fold compared to those synthesized without a cross-linker. The synthesized PEMs with degrees of grafting at 45% and 67% demonstrated superior proton conductivity, outperforming various alternatives, including commercial Nafion samples. Specifically, these cross-linked membranes exhibited promising proton conductivity values of 93.7 and 139.1 mS cm^–1^, respectively. This work highlights the potential of radiation-induced RAFT-mediated polymerization in carrying out cross-linking reactions as an efficient pathway for designing well-defined high-performance PEMs, offering enhanced homogeneity and conductivity compared to existing literature counterparts.

## 1 Introduction

Fossil fuels currently fulfill approximately 80% of the global energy demand, but their utilization raises concerns due to detrimental emissions impacting the environment and human health. In addition to these well-established issues, fossil fuels are under scrutiny for their finite nature and contribution to environmental problems such as global warming.^1,2^ The escalating energy demand, coupled with diminishing fossil fuel reserves, has spurred a growing interest in alternative, renewable energy sources. Extensive research efforts have been directed toward developing sustainable energy systems in recent years. Hydrogen, as one of the most abundant elements globally, presents itself as a viable alternative for clean energy in fuel cell systems. In stark contrast to the 15–35% energy conversion efficiency of fossil fuels, fuel cells boast an impressive 80% conversion efficiency. The versatility of fuel cells extends their application to various devices, including heavy land vehicles, mobile phones, laptops, and even aircraft. Consequently, the imperative to advance fuel cell technologies remains paramount for societal well-being.^3−5^

Fuel cells offer advantages such as high efficiency, quiet operation, modular structures, compatibility with a wide range of fuel types, low emissions, high reliability, ease of installation, and efficient energy conversion and cogeneration. However, challenges such as high costs and insufficient performance/durability hinder the widespread adoption of fuel cells as replacements for existing energy sources.^6,7^ To address these challenges, extensive research is ongoing worldwide to overcome obstacles and innovate new fuel cell membrane technologies. The widely adopted Nafion membrane, developed by DuPont, possesses superior properties, including high chemical, thermal, and mechanical stability due to its fluorinated nature, along with excellent proton conductivity. Despite these attributes, Nafion suffers from drawbacks, most notably its high cost and challenges associated with its synthesis, such as the intricate fluoride chemistry required. Additionally, issues like proton carrier capacity loss at high temperatures and high methanol permeability underscore the need for alternative membranes.^8,9^

Among the diverse strategies employed in the development of Polymer Electrolyte Membranes (PEMs) as alternatives to Nafion, radiation-mediated graft copolymerization emerges as a prevalent method. This approach utilizes a fluorinated polymer film, including polytetrafluoroethylene (PTFE), polyvinylidene fluoride (PVDF), poly(tetrafluoroethylene-*co*-hexafluoropropylene) (FEP), and poly(ethylene-*alt*-tetrafluoroethylene) (ETFE), as the primary backbone. Ionizing radiation is applied to facilitate the grafting of a monomer possessing potential proton carrier properties onto these polymers, with commonly chosen functional groups such as sulfonic acid and carboxylic acid, aiming to introduce proton conductivity.^10−13^ Styrene, an economical monomer, is frequently grafted onto fluorinated polymer films, and subsequent sulfonation provides a sulfonated fluorinated membrane.^3^ Among these fluorinated polymers, ETFE has gained prominence due to its high mechanical strength, radiation stability, and efficient grafting at low doses.^14,15^ To optimize membrane properties, especially the length and frequency of grafted polystyrene chains, this study adopts the reversible addition–fragmentation chain transfer (RAFT) polymerization method—a controlled radical polymerization technique—instead of the conventional free-radical polymerization method widely applied in the literature.^16−19^

RAFT polymerization emerges as a potent tool for precisely forming macromolecular building blocks, enabling the construction of well-defined nanoscale structures.^20^ Its utility extends across various applications, notably in life sciences^21^ such as bioimaging, drug delivery, and cancer therapy, as well as in catalysis^22,23^ and fuel cell.^18,24^ Various strategies have been explored for developing advanced PEM architectures, encompassing grafted copolymers, random copolymers, block copolymers, and cross-linked polymers, all contributing to the understanding of morphology’s impact on proton transfer efficiency. These explorations highlight the relationship between PEM structure and the efficiency of proton transport. The effective transfer of proton species across PEMs, whether in hydrous or anhydrous systems, relies on various factors, including chemical structure, polymer architecture, chain conformation, functional groups, chain flexibility, and side chain acidity. These factors collectively contribute to shaping the morphology of PEMs.^25,26^ The superior uniformity in chain length and architectural homogeneity achieved through the RAFT mechanism, in contrast to conventional free-radical polymerization methods, offers a pathway to establish a more uniform morphology. This, in turn, results in uninterrupted, stable and enhanced proton conductivity.^16,18^

To realize a high-performance proton exchange membrane (PEM), a careful optimization of mechanical, chemical, thermal, and proton conductivity properties is essential. While membrane cross-linking improves mechanical, chemical, and thermal performances, it concurrently limits proton conductivity by restricting chain movements. The degree of cross-linking can be precisely regulated during radiation-induced polymerization using cross-linkers.^27,28^ This control allows for the fine-tuning of membrane properties, balancing factors such as water uptake and proton conductivity with chemical, mechanical, and thermal properties. Numerous instances in the literature highlight the application of radiation-mediated grafting under conventional polymerization conditions, utilizing cross-linkers to prepare cross-linked PEMs.^27−30^ Specifically, the use of divinylbenzene (DVB) as a cross-linker is prevalent in the preparation of radiation-induced grafted membranes for fluoropolymers. Despite some negative impacts on water uptake and proton conductivity compared to alternative methods, the positive influences on properties like mechanical strength and thermal/chemical stability make DVB a commonly employed cross-linker.^30,31^

In a previous investigation, radiation-induced RAFT-mediated grafting demonstrated the production of high-performing ETFE PEMs with remarkable proton conductivity, reaching up to 148.2 mS cm^–1^.^16^ Nonetheless, there is a recognized need for improvement in performance criteria, particularly in terms of chemical, mechanical, and thermal properties. This present study aims to advance the synthesis of PEMs by employing radiation-induced controlled RAFT polymerization to graft polystyrene onto cost-effective ETFE films, incorporating a cross-linking agent (DVB) for the first time. Anticipated outcomes involve achieving structural control and homogeneity through the RAFT mechanism, coupled with the enhancement of optimal physicochemical performance through cross-linking. These combined effects are expected to yield PEMs with distinctive and superior properties compared to conventionally synthesized counterparts. Despite a reduction in proton conductivity attributed to DVB usage, the substantial increase in chemical stability underscores the potential of this novel cross-linked PEM type. The findings provide valuable insights into well-defined, cross-linked PEMs, holding promise for future advancements in fuel cell technologies.

## 2 Experimental Section

### 2.1 Materials

The ETFE polymer film with a thickness of 25 μm (Tefzel 100LZ) was generously provided by the Paul Scherrer Institute, PSI, Switzerland. Styrene (S, Aldrich, 99%) underwent deinhibition through percolation using a column packed with activated basic alumina. Reagent-grade toluene (Aldrich), divinylbenzene (DVB, Aldrich), dichloromethane (Merck), chlorosulfonic acid (Riedel-de Haën), NaOH (Fluka), and H_2_SO_4_ (Merck) were employed as received. The RAFT agent, 2-(dodecylthiocarbonothioylthio)-2-methylpropionic acid (DMMAT, Aldrich, 98%) was utilized in this study. Literature confirms the suitability of this RAFT agent for the controlled polymerization of styrene, and it is commercially available.^32^ All other reagents were procured from Sigma-Aldrich and used in their as-received state.

### 2.2 Preparation of ETFE-*g*-PS Films and ETFE-*g*-PSSA Membranes

In the standard grafting procedure, predetermined amounts of monomer (styrene), RAFT agent (DDMAT), and cross-linker (DVB) were dissolved in toluene. Once complete dissolution of the reactants was achieved, the stock solution was divided into aliquots and transferred to sample glass vials. Subsequently, ETFE film (approximately 2 cm × 2 cm dimensions, ≈0.1 g) was introduced into the vials as the grafting substrate. The vials were sealed with rubber septa and purged with nitrogen gas to remove oxygen. The samples were then exposed to a ^60^Co gamma irradiator at ambient temperature (^60^Co, 1 kGy/h, SANAEM, Sarayköy, Ankara), undergoing various radiation doses. Periodically, samples were extracted from the irradiator to assess reaction kinetics and washed by shaking in toluene until constant weight. After all the nongrafted polymer was removed from the substrate, the films were subsequently dried in the oven to constant weight. The degree of grafting (DG, wt %) was calculated gravimetrically using the initial mass of nongrafted ETFE and the final mass of grafted ETFE copolymer film.

In the pursuit of optimizing experimental conditions, variations were introduced by altering the radiation dose, DVB concentration, and monomer content. The radiation dose spanned from 0.7 to 11.3 kGy to discern its impact. Amidst concentrations ranging from 10% to 80%, 30% (v/v) was identified as the optimum for styrene, a ratio retained in experiments where DVB concentrations were adjusted to 3.5%, 5%, and 10% (v/v). To elucidate the influence of DVB, a subset of experiments was conducted under optimal conditions but without the presence of DVB. The [S]/[RAFT] molar ratio of 700 was consistently applied for the grafting of polystyrene (PS) from ETFE, both in the absence and presence of the cross-linker.

**Figure 1:**
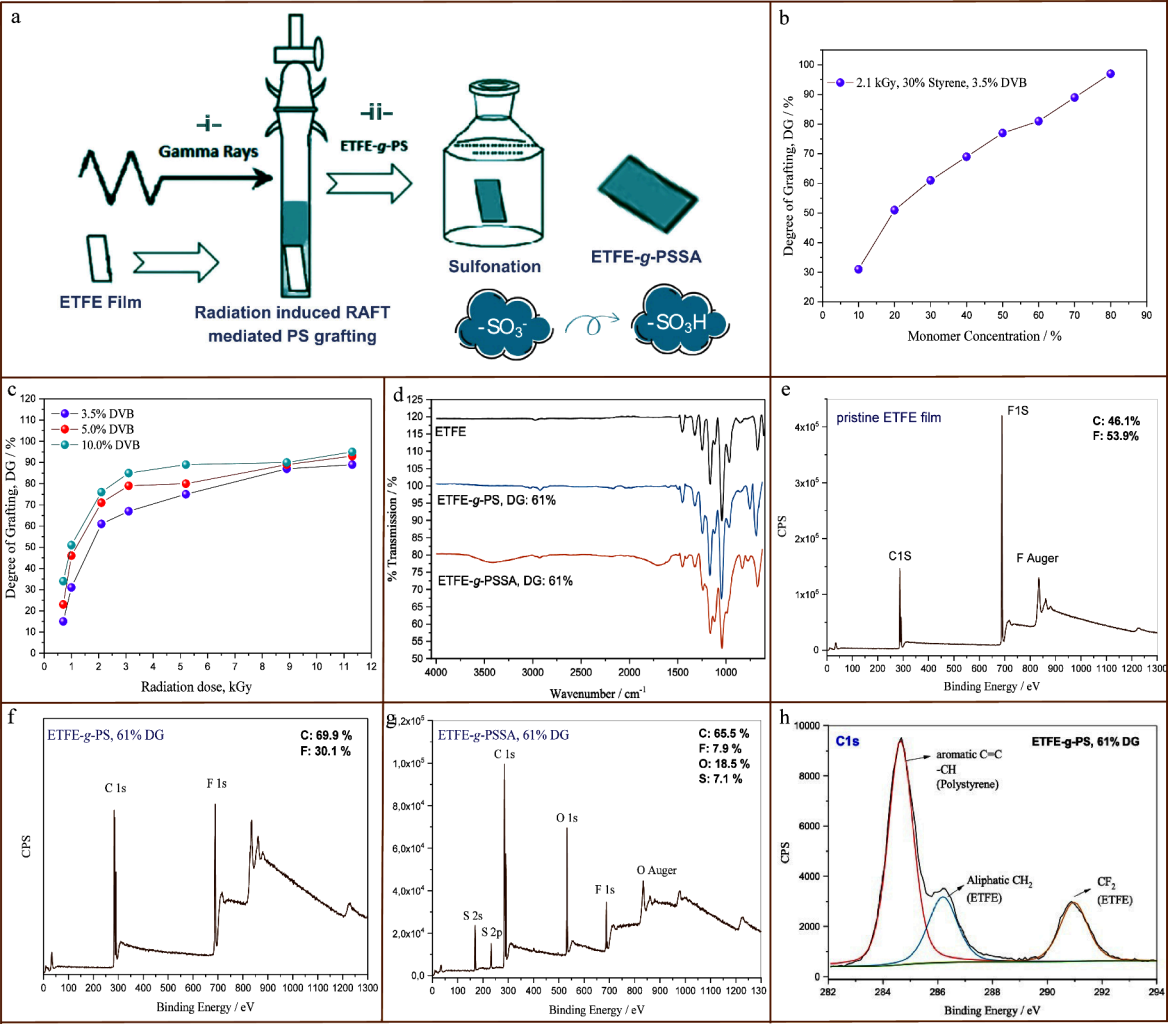
(a) Schematic representation of the radiation-induced RAFT-mediated grafting step (i) and subsequent sulfonation of the grafted film (ii) to yield a proton exchange membrane. (b) Degree of grafting (%) versus monomer concentration (%) for the radiation-induced grafting of styrene (30%, v/v) from ETFE film in the presence of cross-linker (DVB, 3.5%) and RAFT agent (DDMAT) at an absorbed radiation dose of 2.1 kGy. (c) Degree of grafting (%) versus absorbed radiation dose (kGy) at three different DVB concentrations (3.5%, 5%, 10%, v/v) with a styrene concentration of 30% (v/v). [St]/[DDMAT] = 700, ETFE (0.01 g), solvent:toluene. (d) ATR-FTIR spectra of nongrafted (pristine) ETFE, 61% PS grafted ETFE film (ETFE-*g*-PS) and the sulfonated membrane of the same film (ETFE-*g*-PSSA) (from top to bottom). (e–g) Surface wide-scan XPS spectra of (e) nongrafted ETFE, (f) 61% PS grafted (ETFE-*g*-PS) film, and (g) sulfonated membrane of the same film (ETFE-*g*-PSSA). (h) Core-level C 1s XPS spectra of ETFE-*g*-PS (DG: 61%).

The resulting PS grafted ETFE films (ETFE-*g*-PS), exhibiting varying degrees of grafting (DG), underwent multiple washes with toluene to eliminate surface contaminants. These ETFE-*g*-PS films were sulfonated using 10% vol chlorosulfonic acid in dichloromethane for 2 h at room temperature. Subsequently, hydrolysis in a 1 M NaOH solution for 2 h and reprotonation in a 1 M H_2_SO_4_ solution for 3 h yielded ETFE-*g*-poly(styrene sulfonic acid) (ETFE-*g*-PSSA) membranes, utilized as PEMs. The synthesis steps were illustrated in Figure 1a. Pre- and postsulfonation characterizations were conducted on the samples, followed by the preparation of larger membranes under optimally selected conditions with a surface area of approximately 5 cm^2^ for proton conductivity measurements.

### 2.3 Attenuated Total Reflectance Fourier Transform Infra-Red (ATR-FTIR) Spectroscopy

Using PerkinElmer Spectrum One FT-IR spectrometer and ATR module, the IR spectra of the pristine ETFE film, PS-grafted ETFE films and the sulfonated membranes in the range of 400–4000 cm^–1^ were measured. 32 scans were taken and analyzes at 4 cm^–1^ resolution.

### 2.4 X-ray Photoelectron Spectroscopy (XPS)

X-ray photoelectron experiments utilized a monochromatized Al Kα X-ray source from a Thermo spectrometer, employing an X-ray spot size of 400 μm. Thirty eV and 200 eV pass energies were applied during the survey and region scans, respectively. Binding energies (BEs) were referenced to the C 1s peak at 285 eV, and surface elemental compositions were determined across the energy range of 0–1000 eV.

### 2.5 Atomic Force Microscopy (AFM)

Surface morphology changes were investigated using a MultiMode Veeco AFM with a Nanoscope 9.1 controller (Bruker) in tapping mode in air.

### 2.6 Scanning Electron Microscopy (SEM) Imaging and Energy Dispersive X-ray (EDX) Mapping

A FEI Quanta 200FEG microscope captured SEM images at various magnifications. Samples were affixed to an SEM stub with carbon tape and coated with Au (approximately 5 nm) before imaging. Utilizing the Supra 35VP Leo SEM EDAX device with a 15 kV accelerating voltage, the sulfur atom profile in the cross sections of the samples was investigated. The samples were subjected to freezing in liquid nitrogen and subsequently broken to obtain a cross-section for analysis via SEM EDX.

### 2.7 Contact Angle Measurements

Water contact angles of ETFE, ETFE-*g*-PS, and ETFE-*g*-PSSA films were determined using a Krüss DSA100 contact angle goniometer (Krüss GmbH, Germany) at room temperature to assess changes in hydrophobicity/hydrophilicity characteristics. Four contact angles were measured for each sample, and an average value was calculated.

### 2.8 Thermogravimetric Analysis (TGA)

The thermal degradation properties of the polymers were explored employing a PerkinElmer PYRUS Thermogravimetric Analyzer and TA Instruments DMA (Pyris 1 TGA). The analyses were conducted under an N_2_ atmosphere, spanning a temperature range of 25–700 °C, with a heating rate of 10 °C/min. The thermal stability of films and membranes was assessed by examining TGA curves and the first derivatives derived from these curves.

### 2.9 Ion Exchange Capacity (IEC)

The theoretical ion-exchange capacities were calculated as shown in the eq 1, considering that one sulfonic acid group is attached to each styrene unit. Here, DG indicates the degree of grafting, M_St_, is the molar mass of styrene (104.15 g mol^–1^), and M_SSA_ indicates the molar mass of styrene sulfonic acid (184 g mol^–1^).
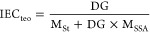
1

To calculate the experimental ion exchange capacities, three samples for each grafting percentage were immediately weighed after drying in the furnace at 60 °C for 1 day. The dry membrane masses were found. Then, the films placed in 10 mL of 1 M NaCl solution were kept for 24 h, and the ion exchange was completed. Titration was performed using standardized 0.01 M NaOH, and experimental ion exchange capacities were calculated using the following equation:
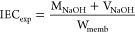
2where M_NaOH_ given in the equation is the molarity of NaOH used in titration, V_NaOH_ is the volume of NaOH used, and W_memb_ is the membrane mass.

Membranes are highly hygroscopic due to the presence of the sulfonic acid groups. Therefore, it would be inaccurate to calculate the percentage of sulfonation gravimetrically. Instead, it is more accurate to calculate the ratio between the experimental ion exchange capacity, and the theoretical ion exchange capacity.

### 2.10 Water Uptake or Swelling Measurements

The water uptake, φ_*w*_ is found as the mass of water absorbed by the membrane divided by the dry weight of the membrane, according to the following equation.
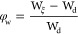
3

In this equation, *W*_ξ_ is the weight of the water-swollen membrane and *W*_*d*_ is the dry weight of the vacuum-dried membrane at 80 °C.

The hydration number, λ, of the membrane is the number of water molecules per sulfonic acid site and defined according to the following eq 4:^31^
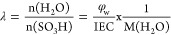
4

### 2.11 Electrochemical Impedance Spectroscopy (EIS) test

Impedance measurements were conducted using the Solartron 1260 Frequency Response Analyzer (FRA) and the Solartron 1287 Electrochemical Interface. For conductivity measurements, a planar four-electrode BekkTech Conductivity cell was employed. Membranes with a surface area of 5 cm^2^ were utilized, and measurements were taken at 25 °C under 100% humidity.

### 2.12 Chemical Stability Test

For the assessment of chemical stability in synthesized samples, the method outlined in literature was followed.^33^ Membranes, approximately 0.5 × 0.5 cm in size, underwent vacuum oven-drying, followed by weighing. Subsequently, they were immersed in pure water until equilibrium water absorption was achieved. The samples were then placed in glass bottles containing a 3% H_2_O_2_ aqueous solution (v/v) and maintained at 60 °C for varying durations. After each incubation period, a sample was withdrawn, reimmersed in distilled water, and agitated for 24 h. In the final step, the rinsed membranes were vacuum oven-dried overnight and reweighed. The mass losses of the membranes were calculated by comparing the initial and final mass measurements.

## 3 Results and Discussion

### 3.1 Optimization of Grafting Conditions and Structural Characterizations

Fluorinated, highly crystalline polymers like ETFE are insoluble in common solvents or monomers and they exhibit minimal or no swelling behavior. The radiation-induced grafting of such polymers follows the “grafting front mechanism” proposed by Chapiro in 1962.^34^ According to this mechanism, polymerization initiates on the surface to be grafted and progresses through the cross-section. The grafted surface layers swell in the polymerization solution, facilitating the diffusion and grafting of monomers into the inner layers. Radicals formed on the substrate during irradiation allow for grafting across the entire polymer cross-section. This mechanism, effective in the presence of a cross-linker, achieves simultaneous grafting and cross-linking, yielding membranes with desired mechanical and thermal properties.^27,28^

Polystyrene, commonly employed in fuel cell studies for its promising sulfonation capacity, faces challenges in chemical stability, prompting the exploration of alternative solutions. Cross-linking is a practical approach to enhance the chemical and mechanical strength of polystyrene. The addition of cross-link reactions to the grafting process leads to improved chemical, thermal, and mechanical properties at the expense of reduced chain movements and, consequently, proton conductivity. In this study, DVB was employed to enhance the physicomechanical properties of grafted films through cross-linking, aligning with previous research. An innovative approach involves grafting in the presence of DVB for the first time via the RAFT mechanism. Carrying out the entire reaction, i.e., grafting and cross-linking, under well-defined conditions via RAFT potentially offers better performance in membrane properties, such as proton conductivity, compared to conventional mechanisms, owing to promising uniform grafting characteristics throughout the entire cross-section of the substrate.

Optimizing grafting conditions is crucial to striking a balance between enhancing and compromising the characteristics of the grafted samples. We varied radiation dose and concentrations of styrene and DVB, as summarized in Table S1 of the Supporting Information. To better illustrate the trends observed in the conducted experiments, the data in this table has been presented in related figures. Figure 1(b) depicts the change in the degree of grafting (DG) of ETFE film in the presence of the DVB cross-linker and DDMAT RAFT agent, depending on the monomer concentration (entities 3–10 in Table S1). In Figure 1(c), the degrees of grafting are presented as a function of absorbed radiation dose, separately for three DVB concentrations. Examining the results presented in Figure 1(b), it is evident that the DG increases with the monomer concentration, ranging from 31% to 97% with monomer variations between 10% and 80%. Higher DG values result in more styrene groups obtained in grafting to carry protons following sulfonation. While this increase in DG is favorable for enhancing membrane properties like proton conductivity, water uptake capacity, and ion exchange capacity, it also brings potential disadvantages. At higher DG values (approximately beyond 60%), membrane integrity begins to deteriorate, and polystyrene’s fragility becomes apparent, leading to phase separations and cloudy regions. As shown in Figure 1(b), monomer concentrations above 30% are deemed unnecessary in practical applications due to the excessively high DG achieved, making the use of less monomer preferable from an economic standpoint.

In Figure 1(c), the results of grafting conducted with 30% (v/v) styrene in the presence of three different DVB concentrations (3.5%, 5%, and 10%) are presented as a function of radiation dose, representing reaction time. The figure illustrates that the DG experiences a rapid increase at low radiation doses for all three DVB concentrations. Beyond approximately 3 kGy radiation dose, the increase in DG slows down significantly, reaching a stabilization point. Over time, both monomer and DVB in the reaction medium are consumed. The DG remains relatively constant after a certain radiation dose, signifying an equilibrium value. Increasing the DVB concentration results in an anticipated increase in monomer conversion, leading to more polymer being grafted onto ETFE. As indicated in Figure 1(c), an ETFE-*g*-PS copolymer with a 31% degree of grafting was achieved at only 1 kGy irradiation with 3.5% DVB. Since the optimal monomer ratio was chosen as 30%, the 1 kGy radiation dose, where a sufficient degree of grafting (around 30%) was reached, is deemed the optimum radiation dose. Consequently, unless otherwise specified, all subsequent experiments in this study were conducted with 30% styrene and 3.5% DVB, with a monomer/RAFT agent molar ratio of 700 and a radiation dose of 1 kGy. It is essential to note that the same degree of grafting could not be consistently achieved in repeated experiments, and the synthesized films were not of sufficient size for all tests, leading to the use of films with different DGs in the subsequent characterizations of this study. Additionally, while the grafted films are denoted as ETFE-*g*-PS, it should be acknowledged that the DVB cross-linker has also contributed to the composition of PS chains through the formation of cross-links. For convenience, the notation ETFE-*g*-PS is preferred for the graft copolymer structures in the later parts of this study. In the context of Table S1, as expected, a lower DG was obtained from the synthesis conducted in the absence of DVB (entity 29), and these samples were reserved for postmembrane property tests.

The synthesized samples underwent initial characterization using the FTIR method (Figure 1d). The FTIR spectra below depict pristine ETFE, 61% PS grafted ETFE, and the membrane obtained by sulfonating the ETFE-*g*-PS film. Samples with degrees of grafting below 50% were retained for further testing due to their critical importance in determining membrane properties, while samples with higher DGs (61% for FTIR) were utilized in structural characterizations. In the ATR-FTIR spectrum of ETFE, weak peaks at 2976 and 2880 cm^–1^ reveal characteristic asymmetric and symmetrical aliphatic −CH_2–_ stretching. The severe peaks in the FTIR spectrum originating from −CH stretching are mitigated by the electron-withdrawing property of F atoms in the ETFE structure, enhancing the polarity of the partial −CH bonds.^35^ Strong characteristic peaks of −CF_2_ stretching and −CH_2_ deformations are observed between the 1500–500 cm^–1^ region, with wagging and scissoring deformation peaks of CH_2–_ at approximately 1453 and 667 cm^–1^. Sharp peaks from −CF_2_ groups appear in the 1000–1300 cm^–1^ range.^16,36^

In the spectrum of the 61% PS grafted ETFE-*g*-PS sample, characteristic peaks of aromatic polystyrene are observed in the 2700–3200 cm^–1^ range. The C–H deformation bands of the monosubstituted benzene ring appear at approximately 696 and 756 cm^–1^, providing evidence of PS binding to the structure.^37^ The ETFE-*g*-PSSA copolymer spectrum resulting from sulfonation reveals a wide absorption band between 3000 and 3600 cm^–1^, attributed to −OH groups in water molecules held by sulfonic acid groups, indicating a transition to a hydrophilic state. The broadband in the 1600–1700 cm^–1^ range is due to −OH structures in the sulfonic acid groups. Stretching peaks of the sulfated benzene ring and vibration peaks from the −SO_3_ groups are observed at 1004 and 1135 cm^–1^, respectively. C–H deformation peaks in the disubstituted benzene ring appear at 832 and 774 cm^–1^.^38,39^ These spectroscopic results collectively confirm the successful execution of the synthesis.

XPS serves as a primary method for surface characterization. The XPS results of nongrafted ETFE, 61% PS grafted film (ETFE-*g*-PS), and the sulfonated membrane with the same degree of grafting (ETFE-*g*-PSSA) are presented in Figure 1e-g, respectively. In the wide-range surface scan spectrum of the ETFE film, the F 1s peak is observed at around 689 eV binding energy (BE), and the C 1s peak appears near 290 eV (Figure 1e).^16,40^ The atomic percentages calculated from F 1s and C 1s peaks indicate their presence in the structure at approximately 53.5% and 46.1%, respectively. The expected molar ratios of ethylene and tetrafluoroethylene monomers for consecutive ETFE copolymers should be 50%–50%, resulting in an equal amount of C and F elements. However, in commercial ETFE polymers, this ratio can vary, typically ranging between 60 and 40%.^41^ The ethylene-tetrafluoroethylene mole ratios for the ETFE sample used in this study were found to be approximately 46% and 54%, respectively.

Examining the XPS spectrum of the 61% PS grafted ETFE-*g*-PS film (Figure 1f) reveals a substantial increase in the C element due to the addition of PS chains, increasing from 46.1% to 69.9%. Consequently, the percentage of F element decreases to 30.1%. Upon analyzing the ETFE-*g*-PSSA membrane with a 61% degree of grafting obtained by sulfonation, the addition of O (15.7%) and S (4.3%) atoms to the structure is observed (Figure 1g). O atoms appear at approximately 532.2 eV, while S atoms appear at 168.5 eV, confirming the presence of sulfonic acid (−SO_3_H) groups.^16^ The percentage of F atoms decreases to 7.9%, and C decreases to 65.5%. Despite the addition of abundant −SO_3_H groups, the relatively small decrease in C atoms suggests enrichment of the surface with grafted chains, possibly pushed through free volume regions via electrostatic interactions. XPS not only provides elemental composition but also offers insights into the chemical environment of the elements. Core-level C 1s and O 1s spectra were examined in detail. In the ETFE-*g*-PS sample with a 61% degree of grafting, the C 1s spectrum in Figure 1h shows two peaks corresponding to CH_2_ and CF_2_ groups of ETFE, along with a third peak at a higher binding energy, indicating the presence of aromatic C=C and −CH structures of grafted PS.^42^ The O 1s spectrum of the ETFE-*g*-PSSA membrane presented in Figure S1 of Supporting Information, exhibits two components corresponding to S=O groups (532 eV) and O–H groups (533.3 eV). The observed ratio of these peaks (approximately 2:1) aligns with the expected structure of −SO_3_H groups.^36^

XPS analyses were also conducted for ETFE-*g*-PS films with varying DGs and their sulfonated membranes, and the surface elemental composition for each sample is provided in Table S2 of the Supporting Information. Examination of the values in Table S2 reveals a significant increase in the percentage of C atoms in the structure and a corresponding decrease in F atoms with an increase in the degree of grafting of polystyrene to ETFE, as anticipated. In the ETFE-*g*-PS sample with an 87% degree of grafting, the presence of S atoms originating from the sulfur-containing RAFT chain-end moieties is observed, indicating the involvement of RAFT polymerization in the synthesis mechanism. In the sulfonated ETFE-*g*-PSSA samples, S and O atoms are clearly detected in the XPS results due to the addition of −SO_3_H groups to the structure, with the amounts of these elements increasing with the degree of grafting.

**Figure 2:**
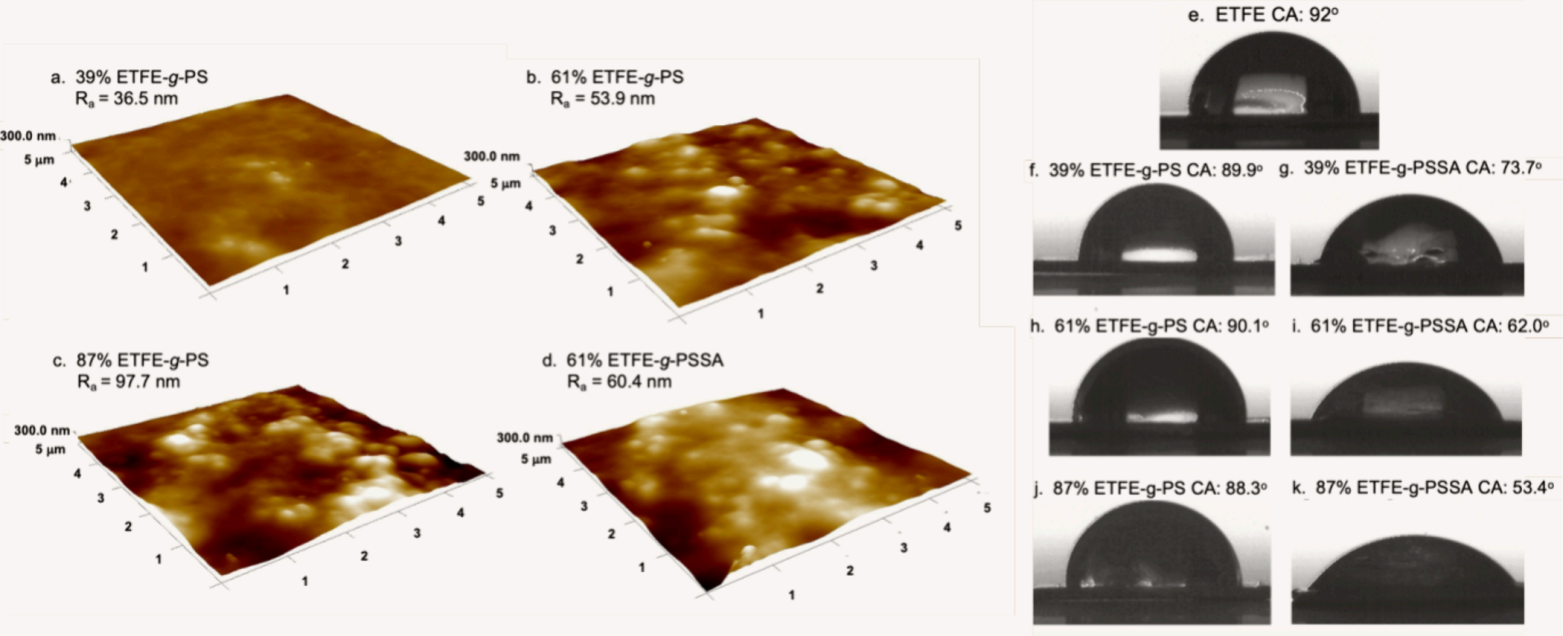
AFM images and roughness values (Ra) of PS grafted films (ETFE-*g*-PS) with varying DGs: (a) 39%, (b) 61%, and (c) 87%, along with (d) a sulfonated membrane (ETFE-*g*-PSSA) derived from a 61% PS grafted film. Water contact angle (CA) images of pristine ETFE are shown in (e), while (f), (h), and (j) represent ETFE-*g*-PS with 39%, 61%, and 87% degrees of grafting, respectively. (g), (i), and (k) depict the corresponding sulfonated ETFE-*g*-PSSA membranes.

The AFM analysis of PS grafted films (ETFE-*g*-PS) with varying DGs (39%, 61%, and 87%), and a sulfonated membrane (ETFE-*g*-PSSA) derived from a 61% PS grafted film was conducted to examine surface morphologies. AFM images and calculated roughness values (Ra) are presented in Figure 2a-d. The results reveal an increase in surface roughness due to grafting, with a higher DG corresponding to a greater increase in roughness. Comparatively, pristine ETFE film, as shown in Figure S2 of Supporting Information, exhibits the lowest Ra value of 14.3 nm. The inert nature of ETFE with respect to solvents is altered upon PS grafting, rendering it swellable in compatible solvents. This transformation results in an increase in size and warping on the film surface. The grafting process itself contributes to morphological changes too. Comparing AFM images of different PS grafting percentages, a clear trend of increased roughness with higher DG is observed. In the AFM image of the 87% grafted ETFE-*g*-PS sample, distinct PS clusters become evident, signifying surface heterogeneity at very high DGs. This heterogeneity is visually noticeable in samples with DGs exceeding 60%. Sulfonation induces a minor change in surface morphology and roughness, with Ra increasing slightly from 53.9 to 60.4 after sulfonation of the grafted ETFE-*g*-PS film with 61% DG. This suggests that both ETFE and PS undergo limited morphological changes during the sulfonation reaction.

Contact angle measurements were employed to validate the surface properties of grafted films and sulfonated membranes. Figure 2e-k illustrate that the water contact angle of pristine ETFE remains nearly constant across all degrees of grafting in PS grafted ETFE films. The water contact angle (CA) of polystyrene-grafted films (ETFE-*g*-PS, left column) does not exhibit significant changes with varying degrees of grafting, aligning with prior literature.^43^ Given the hydrophobic nature of both ETFE and PS structures, a substantial alteration in CA is not anticipated postgrafting. However, a notable decrease in CA is observed due to the introduction of hydrophilic characteristics after sulfonation. Scherer et al. reported a decrease in water CA to 32° after sulfonation for a DG of 82% in non-cross-linked membranes, while cross-linked structures exhibited a less pronounced decrease due to limited chain segment mobility.^44^ In our study, water CA values decreased with increasing degrees of grafting in sulfonated membranes, reaching 53.4° at a DG of 87%, consistent with existing literature.

**Figure 3:**
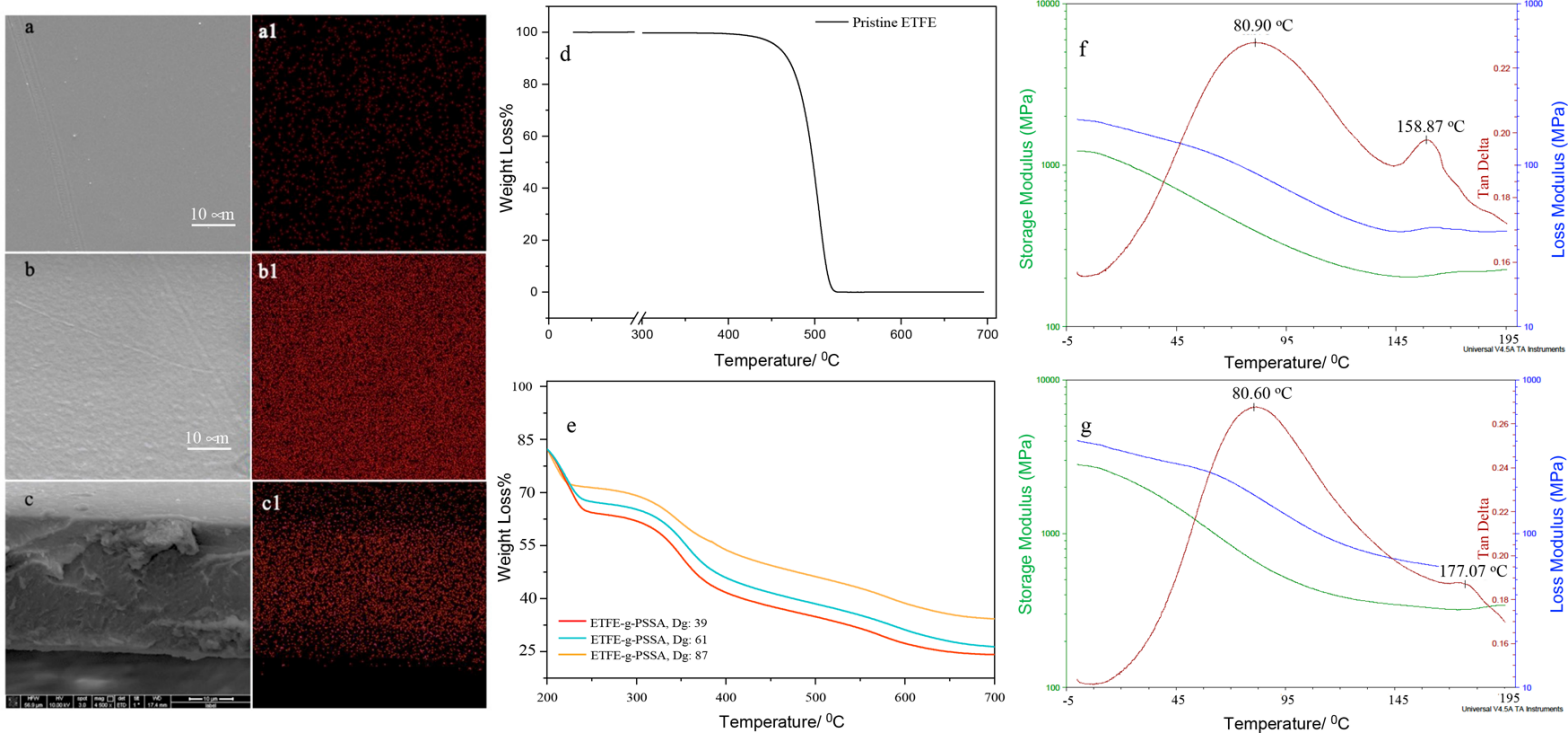
SEM images of (a) the surface of PS grafted ETFE film (ETFE-*g*-PS, DG: 61%), (b) the surface of the sulfonated membrane (ETFE-*g*-PSSA, DG:61%), and (c) the cross-section of the sulfonated membrane (ETFE-*g*-PSSA, DG: 61%). Corresponding SEM-EDX distributions of S atoms are illustrated in a1, b1, and c1. TGA thermograms for (d) pristine ETFE and (e) ETFE-*g*-PSSA samples with different degrees of grafting: red curve for DG: 39%, blue curve for DG: 61%, and yellow curve for, DG: 87%. The changes in tan δ, storage modulus, and loss modulus of ETFE-*g*-PS films with DGs of (f) 39% and (g) 87% against temperature.

For efficient proton transmission across the membrane, PS must be grafted not only on the surface but also throughout the inner regions of the membrane. Figures 3a and b present SEM images and SEM-EDX mappings for S elements on the surface of PS grafted ETFE film with a DG of 61% and the sulfonated membrane derived from this film, respectively. In Figure 3a1, the S element mapping shows a limited number of red dots corresponding to S atoms in the RAFT-moieties at the chain ends of PS grafted to ETFE. After sulfonation, a substantial increase in S atoms is observed across the entire surface, exhibiting a homogeneous distribution, indicating uniform grafting and subsequent sulfonation. Successful proton conductivity requires grafting throughout the entire membrane cross-section, allowing protons to be transported through −SO_3_ groups. Cross-sectional SEM and SEM-EDX analysis of the ETFE-*g*-PSSA membrane with a DG of 61% confirm grafting throughout the membrane’s cross-section, as indicated by the significant and homogeneously spread S atoms detected in Figure 3c1. This suggests that grafting occurs uniformly both on the surface and throughout the entire cross-sectional area of the membranes.

TGA is a valuable method for exploring the structure and thermal stability of grafted films. In Figure 3d and 3e, TGA curves of pristine ETFE film and sulfonated membranes (ETFE-*g*-PSSA) with DGs of 39%, 61%, and 87% are displayed. The thermograms reveal that the pristine ETFE film exhibits a single-step degradation profile, initiating at approximately 440 °C, with minimal mass loss up to the main degradation pattern at around 505 °C. In contrast, the TGA results for PSSA grafted membranes showcase a multistep degradation profile. Membranes with varying degrees of grafting exhibit high hydrophilicity due to sulfonic acid groups in their structures. Each membrane experiences roughly a 15% mass loss up to about 200 °C, indicative of significant water absorption by ETFE films post PSSA modification. However, despite the expected increase in water content with the degree of grafting, all membranes exhibit nearly identical water content levels. This inconsistency with the water uptake capacity tests presented later may result from a lack of standard drying before TGA, potentially causing variable evaporation of easily removable water molecules from the structure. Previous studies have noted that the degradation patterns of ETFE-*g*-PSSA membranes are influenced by the drying procedure before TGA measurements.^30^ ETFE-*g*-PSSA membranes display a three-step degradation profile. The initial step, occurring at approximately 250–300 °C, is succeeded by desulfonation, where sulfur oxides separate from the structure. Subsequently, the main degradation pattern of ETFE is observed at around 550 °C. This degradation behavior is consistent with findings in the literature.^16^ In a study by Youcef et al., comparable initial and maximum decomposition temperatures, along with a three-step degradation profile, were reported for cross-linked ETFE-based membranes.^30^ Moreover, pristine ETFE film shows virtually no remaining mass at the end of thermal degradation (Figure 3a). In contrast, sulfonated samples display residual mass, with the quantity increasing proportionally to DG, as anticipated and documented in the literature.^45^ The residual mass percentages of sulfonated samples were 23.8%, 26.2%, and 34.5% for the membranes with 39%, 61%, and 87% DG, respectively.

DMA proves to be an effective method for determining the glass transition temperatures of polymers, with the maximum peak values in the tan δ curves corresponding to the polymer’s glass transition temperature (Tg). In Figure 3f and 3g, it is observed that ETFE-*g*-PS samples exhibit Tg at two distinct temperatures. Pristine ETFE film has a reported Tg of approximately 135 °C.^46^ Polystyrene possesses a Tg around 100 °C, observable in lower temperature ranges up to 50–60 °C, depending on its molecular weight.^47^ The first glass transition temperature, occurring around 80 °C, corresponds to the PS chains grafted into the structure. Both ETFE-*g*-PS films with DGs of 39% and 87% show similar Tg temperatures for PS. The second Tg value at higher temperatures is attributed to the ETFE unit in the structure. While the Tg value of pristine ETFE is about 135 °C, grafting induces an increase in Tg values. The grafting of PS to ETFE limits the mobility of ETFE chains, resulting in an elevated Tg value. The Tg value of ETFE-*g*-PS with 87% grafting (approximately 177 °C) surpasses that of the 39% PS grafted ETFE sample (around 158 °C) since the mobility of ETFE chains is further restricted by increased grafting in the structure.

### 3.2 Evaluation of Membrane Properties of ETFE-*g*-PSSA Membranes

**Figure 4:**
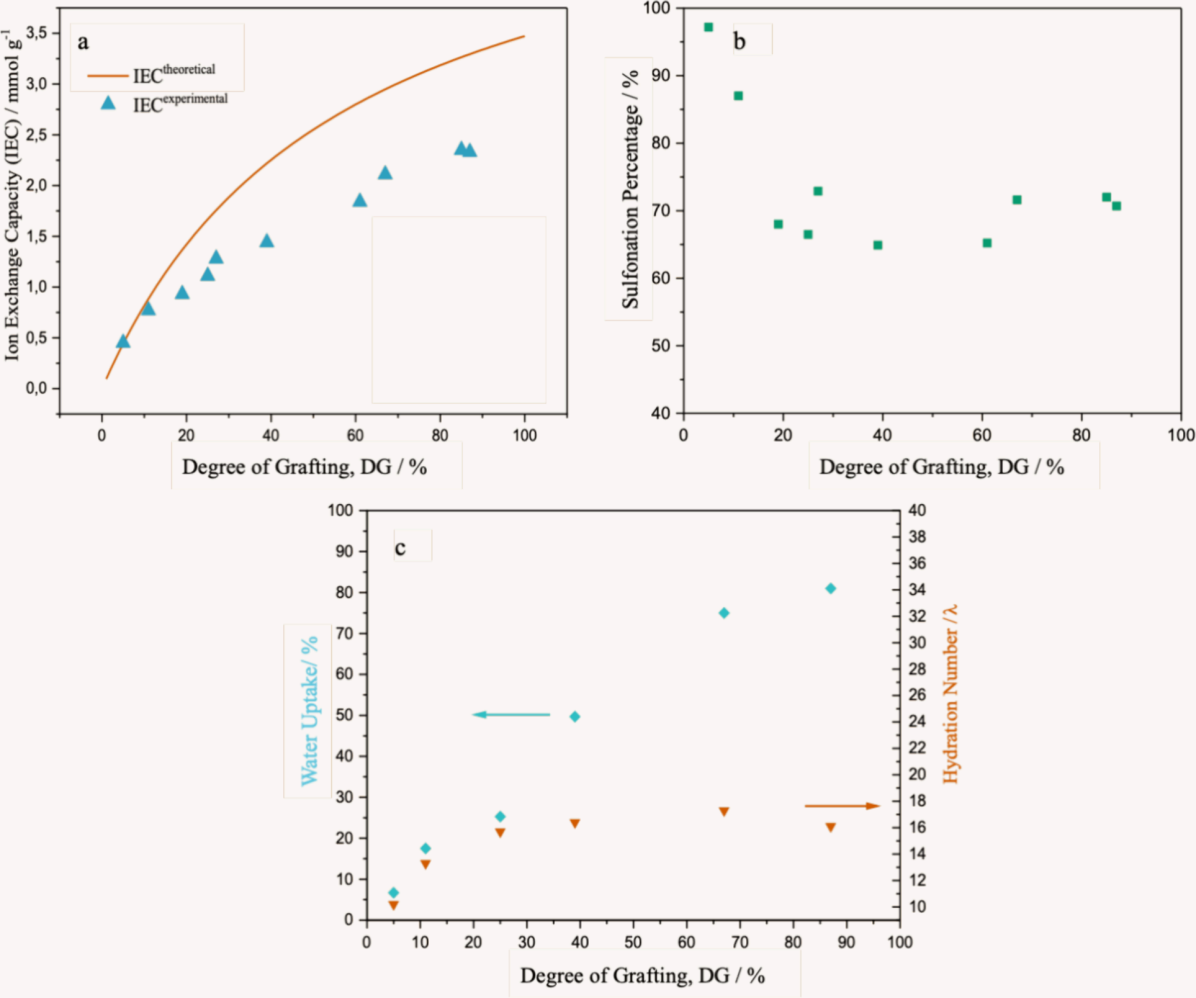
(a) The change of ion exchange capacity (IEC) with the degree of grafting in ETFE-*g*-PSSA membranes (DG: 5–87%). The variation of sulfonation percentage varies as a function of the degree of grafting in ETFE-*g*-PSSA membranes (DG: 5–87%). (c) The water uptake capacity and hydration number of ETFE-*g*-PSSA varying with different degrees of grafting (DGs: 5%, 11%, 25%, 39%, 67%, and 87%).

The ion exchange capacity (IEC) values were determined as a reliable measure of the number of acidic groups with ion exchange capacity in the membrane structures. IEC measurements provide an indirect but effective estimate of proton conductivity.^48^ The calculated IEC values, using the provided equations, were plotted against the degree of grafting, as shown in Figure 4a. Sulfonation percentages were then determined by comparing experimental and theoretical IEC values, and the results are presented in Figure 4b. Upon examination of Figure 4a, it is evident that the experimental IEC and theoretical IEC values are highly compatible at low degrees of grafting. However, this initial agreement weakens with increasing DGs. The sulfonation percentage stabilizes in the range of approximately 60%–70%, as observed in Figure 4b, with some deviations. These deviations could be attributed to the membranes’ potential inability to complete ion exchange while being held in the NaCl solution.^48^ Furthermore, the IEC demonstrates an increasing trend with the degree of grafting, signifying a rise in the number of sulfonic acid groups within the membrane structure as the DG increases. The IEC values achieved through grafting reach levels that are promising for application as a fuel cell membrane. For context, the IEC value of the commercially used Nafion-112 is reported to be 0.91 mmol/g.^49^ In the membranes synthesized in this study, this IEC value could be surpassed with only a 19% grafting. This highlights the potential suitability of the synthesized membranes for fuel cell applications, given their comparable or even superior IEC values compared to established commercial counterparts.

One of the crucial parameters influencing membrane performance is water uptake capacity, providing insights into the membrane’s water absorption and the number of hydrophilic groups present. The hydration number, derived from water uptake capacity, indicates the number of water molecules retained per sulfonic acid group, crucial for proton mobility and membrane conductivity in perfluoro sulfonic acid structures.^50^ As shown in Figure 4c, the hydration number and water uptake capacity values of perfluoro sulfonic acid membranes exhibit an upward trend with an increase in the degree of grafting. This correlation arises from the augmented presence of hydrophilic sulfonic acid groups resulting from grafting. A benchmark for comparison is the Nafion 112 membrane, widely used commercially, boasting a water uptake capacity of 33.5% and a hydration number of 18.^12,31^

Comparison of water uptake capacity, ion exchange capacity (IEC), and hydration number with similar membranes reported in the literature and commercial Nafion samples is presented in Table 1. The water uptake capacity of the membranes synthesized in this study ranges from 6.7% to 81%, showcasing relatively high values. This high-water uptake is attributed to the cross-linked polymeric network’s ability to absorb water throughout the entire membrane cross-section. Notably, despite increased water uptake, the hydration number, indicating the number of water molecules per repeating unit, reaches a maximum of 17.3. Intriguingly, after a certain degree of grafting (around 40%), the hydration value either remains constant or experiences a slight decrease. This contrasts with prior reports on membranes obtained by grafting polystyrene to ETFE film without DVB.^16^ The varying conditions across reported proton exchange membranes (PEMs), including radiation dose rates, absorbed doses, concentrations, and temperatures, contribute to the wide range of reported DG values and membrane properties. However, the values obtained in this study are generally comparable or even superior to those previously reported, especially when compared with commercial Nafion samples.

**Table 1:** Comparison of Our Results with Other Works Reported on ETFE-*g*-PSSA Membranes Synthesized by Radiation-Induced Grafting Method and Commercial Nafion Samples

DG, %	DVB, %	IEC (mmol/g)	Water Uptake, wt %	Hydration number, λ	ref
25^a^	3.5	1.11	26.0	15.5	This study
39^a^	3.5	1.44	51.0	16.0	This study
67^a^	3.5	2.11	77.0	17.0	This study
37^b^	0	1.71	41.0	13.0	(16)
48^b^	0	2.08	64.0	17.0	(16)
25.6	0	1.71	33.9	10.8	(28)
36	0	1.51	30.0	–	(29)
45–55	0	2.15–2.45	–	11.0	(51)
25.8	5	1.71	21.9	7.0	(28)
51.7	10	2.22	14.2	3.55	(31)
30.4	10	1.66	10.3	3.44	(28)
24.1	10	1.74	14.1	4.6	(28)
24.9	20	1.45	7.8	3.0	(28)
Nafion 112	0	0.91	33.5	18.0	(28)
Nafion 105	0	1.00	51.0	28.0	(29)
Nafion 117	0	0.89	37.0	23.0	(29)

a ETFE-g-PSSA in this study by RAFT-mediated grafting of PS in the presence of DVB.

b ETFE-g-PSSA by RAFT mediated grafting of PS without DVB. All the other ETFE-g-PSSA membranes are those synthesized by the conventional radiation-induced grafting method.

The potential of the synthesized membranes to exhibit high proton conductivity was evaluated by determining the proton conductivity value (σ, S cm^–1^) using eq 5.
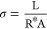
5

In this formula, R (Ω) represents the membrane resistance obtained from impedance measurements, A is the membrane cross-sectional area for current flow (cm^2^), and L is the membrane thickness (cm). Measurements were conducted on approximately 2.5 × 2.5 cm-sized samples. The results are presented in Table 2.

**Table 2:** Membrane Properties Obtained in This Study and Their Comparison with Literature Data and Commercial Nafions^a^

Entity No.	Sample DG, %	DVB, %	Thickness L/μm	Ohmic Resistance (R)/mΩ cm^2^	Conductivity (σ)/mS cm^–1^	ref
1	25^b^	3.5	41.2	317	19.2	This study
2	39^b^	3.5	45.6	99.7	70.9	This study
3	45^b^	3.5	47.7	73.3	93.7	This study
4	67^b^	3.5	53.3	57.9	139.1	This study
5	53^c^	0	46.1	38.9	173.9	This study
6	48^d^	0	47.3	31.86	148.2	(16)
7	36	0	90.0	250.0	43.0	(29)
8	25.6	0	25.0	–	102.0	(28)
9	25.8	5	25.0	95.0	62.0	(28)
10	24.1	10	25.0	–	26.0	(28)
11	24.9	20	25.0	–	16.0	(28)
12	18.2	10	25.0	113.0	41.0	(28)
13	34	0	145.0	130.0	108.0	(29)
14	Nafion 112	0	58.0	86.0	82.0	(31)
15	Nafion 105	0	210.0	220.0	51.0	(29)
16	51.7	10	39.0	83	41.0	(31)
17	45	5	50.0	–	70.0	(51)

a The measurements were conducted at 25°C and 100% humidity.

b ETFE-g-PSSA prepared in this study by RAFT-mediated grafting in the presence of DVB.

c ETFE-g-PSSA prepared in this study by RAFT-mediated grafting in the absence of DVB. This sample was synthesized at a radiation dose of 11.3 kGy.

d ETFE-based PEM by RAFT-mediated grafting in the absence of DVB–literature data. All the other ETFE-g-PSSA membranes were synthesized by conventional radiation-induced grafting method.

Upon examination of the results presented in Table 2, it is evident that the synthesized membranes exhibit promising characteristics. Notably, the 45% and 67% grafted membranes demonstrated higher conductivity than commercial Nafion 112 and Nafion 105 membranes. However, the introduction of DVB resulted in reduced proton conductivity, despite an increase in the degree of grafting (compare entities 4 and 5). Moreover, literature reports indicate a higher proton conductivity of 148.2 mS cm^–1^ at a DG of 47% without using DVB, compared to our data at a similar DG (entity 6 Table 2).^16^ This outcome aligns with expectations, as the presence of a cross-linker like DVB tends to reduce proton conductivity, as reported in previous studies.^27,28^ Cross-linked networks impede mass transfer, limiting proton conductivity, but concurrently enhance the mechanical and chemical strength of the membranes. Striking a balance between these opposing factors is crucial for developing membranes that can truly compete with Nafion, considering both strength and conductivity.

The proton conductivity values of PEMs synthesized through similar methods exhibit a wide range of variation in the literature. This variability can be attributed not only to differences in experimental conditions but also to the use of nonstandardized measurement methods and devices. Nevertheless, the membranes synthesized in this thesis demonstrate high conductivity compared to results obtained in studies employing relatively similar experimental methods. For example, the highest reported proton conductivity of an ETFE membrane with a PS DG of 45% was 70 mS cm^–1^.^51^ In another study, the conductivity of a DVB-cross-linked PS grafted ETFE film with a DG of 51.7% was reported as 41 mS cm^–1^.^31^ Notably, all these previous works employed conventional free radical polymerization rather than RAFT polymerization. The promising outcomes obtained in our study suggest that the well-defined and uniform grafting profile achieved through the RAFT mechanism imparts consistent graft features throughout the membrane. This uniformity enables “uninterrupted” proton conductivity across the entire membrane cross-section.

### 3.3 Chemical Stability of the Membranes

**Figure 5:**
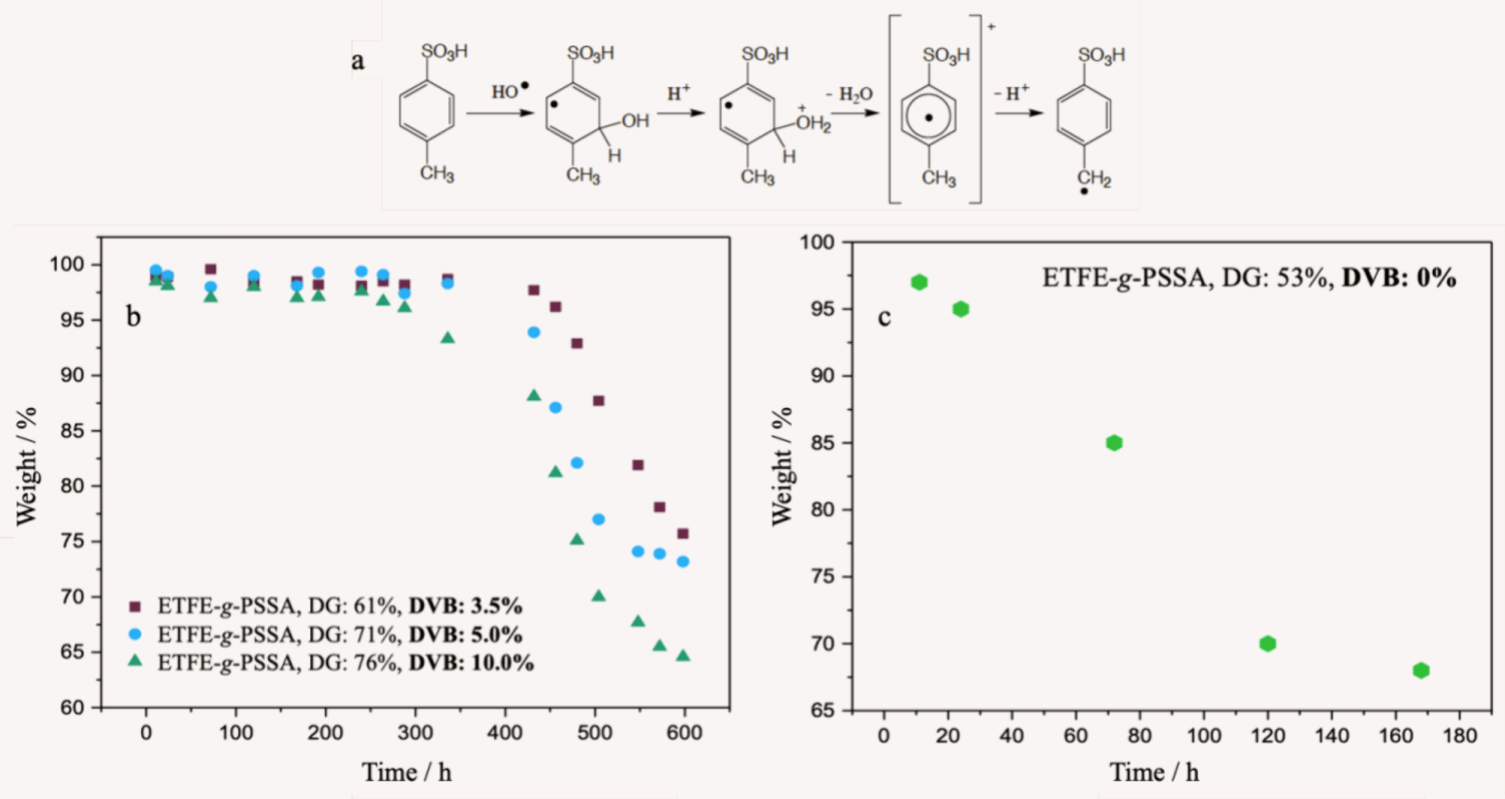
(a) Illustration of the fundamental degradation mechanism of PSSA-based membranes.^52^ (b) Chemical decomposition pathways of ETFE-*g*-PSSA membranes with different degrees of grafting, synthesized using various DVB concentrations at the same radiation dose (2.1 kGy) in a 3% H_2_O_2_ solution at 60 °C. (c) Chemical decomposition pathway of ETFE-*g*-PSSA membrane with a 53% degree of grafting, synthesized in the absence of DVB in a 3% H_2_O_2_ solution at 60 °C.

The degradation mechanism of PEMs based on PSSA generally hinges on the vulnerability of the hydrogen in the α position of the styrene group. In the literature, the products resulting from the degradation reaction of para-toluenesulfonic acid molecules in a highly reactive chemical medium, such as H_2_O_2_, were investigated using Electron Paramagnetic Resonance Spectroscopy (EPR).^52^ It was observed that the alpha-hydrogen on the aromatic ring was highly susceptible to breaking from the structure due to the attack of ˙OH radicals. The degradation mechanism initiated with the rupture of these hydrogen atoms, proceeded with the radicals formed on the main polymer chain, and ultimately led to chain breakage (Figure 5a). While similar degradation mechanisms occur for membranes employed in fuel cell construction, this degradation model may not comprehensively elucidate all the chemical degradation processes that transpire in PSSA-based membranes and how degradation unfolds under fuel cell operating conditions.

Chemical degradation is not the sole problematic factor for Membrane Electrode Assemblies (MEAs); physical factors also contribute to membrane degradation. Under fuel cell operating conditions, it has been determined that membranes undergo thinning, or pinhole-like formations occur in the structure over time.^53,54^ The initial physical properties of membranes play a crucial role in determining their thermal and mechanical stability. Therefore, parameters such as glass transition temperature, elongation at break, and tensile strength are vital for membrane stability. Conventional fuel cell durability tests extend over more than a thousand hours, excluding design and component development. Consequently, fuel cell durability tests are intricate and not conducted randomly. Mechanical tests performed for any polymer may yield meaningless results for a fuel cell membrane due to differences between operating and measuring conditions. Reliable protocols are required for short-term tests to facilitate a swifter evaluation of the membrane’s mechanical strength potential and durability in a fuel cell. Numerous studies in the literature propose various accelerated decomposition protocols (such as relative humidity cycles, start/stop cycles, Fenton reagent, etc.).^55^ One of the most practical and frequently used tests is the Fenton test. In a simplified version of the Fenton test, unlike the standard procedure, Fe^2+^ ions are not added to the medium, and the degradation reaction is carried out under milder conditions. This chemical stability test is commonly employed in the literature by following the steps outlined in the experimental section.^33^ The data obtained from this test are presented in Figure 5b for samples with varying DGs synthesized using different amounts of DVB and applying the same radiation dose.

The radiation dose employed in grafting significantly influences the physical and chemical properties of the membranes. Some degree of degradation in membranes may be observed at high radiation doses. Hence, samples with the same radiation dose were carefully chosen to ensure reliable comparisons in chemical stability tests. To achieve membranes with different DGs at the same radiation dose, samples with varying amounts of DVB (cross-linker) were selected. However, in this scenario, not only the amount of DVB but also the degree of grafting, as a dependent parameter, could change between the samples. As anticipated, an increase in the DVB amount corresponds to an increase in the degree of grafting. In summary, the simultaneous alteration of both the DVB amount and the degree of grafting in the samples used for chemical stability tests poses some challenges in interpretation. Despite these complexities, the results in Figure 5b provide valuable insights. Notably, a decrease in the chemical stability of the samples is observed with an increase in the amount of DVB used, and consequently, with the increase in the degree of grafting. At first glance, it might seem counterintuitive to observe a decrease in chemical stability with an increase in the amount of DVB. However, the increased DVB implies more chain ends and shorter polymer chains between cross-links. Since chain ends are more susceptible to chemical decomposition, it is hypothesized that increasing the amount of DVB facilitates chemical decomposition in the membranes. As mentioned earlier, the optimized DVB amount of 3.5%, determined from the data in Figure 1c, was used consistently throughout the study. An unexpected benefit of using a low DVB amount is the increased chemical stability, as illustrated in Figure 5b. The achievement of high chemical stability with a low DVB amount is a significant advantage.

Nevertheless, it is crucial to highlight that an optimal degree of cross-linking enhances chemical stability, and the use of DVB has an optimum value in this regard. Notably, the absence of DVB causes a substantial decrease in chemical stability. To investigate this, a similar experiment was conducted using a sample synthesized without DVB (Table 2, entity 5), and the results are illustrated in Figure 5c. As depicted, the membrane synthesized without DVB experiences significant chemical decomposition after only around 100 h. In contrast, samples synthesized in the presence of DVB exhibit a similar level of degradation only beyond 400 h, with almost no degradation in the first 300 h. These findings align with previous studies, such as those reported by Nasef et al., which demonstrated an increase in chemical stability in terms of mass loss from 55% (non-cross-linked) to 22% (cross-linked).^54^ It is well-established that the chemical stability of proton exchange membranes is significantly enhanced by cross-linking, provided an optimal level is maintained.^33^ The results underscore that despite the decrease in proton conductivity due to the use of DVB, cross-linking represents a significant advantage due to the substantial increase in chemical stability.

## 4 Conclusions

In this study, we successfully engineered well-defined PEMs through radiation-induced grafting of polystyrene onto cost-effective ETFE films using RAFT polymerization, coupled with the introduction of a cross-linker for the first time. The extensive characterizations conclusively verified the presence of grafted polystyrene chains within the copolymer matrices and the subsequent successful sulfonation process. These results not only provide crucial insights into the advantages of the radiation-induced RAFT polymerization method in terms of structural homogeneity but also highlight the enhanced membrane properties achieved through this approach. Our study revealed promising results in proton conductivity, showing competitive outcomes compared to existing literature. The synthesized PEMs exhibited favorable proton conductivity when compared to various alternatives, including commercial Nafion samples. However, it is imperative to acknowledge the inherent trade-off between proton conductivity and physicochemical stability, underscoring the necessity for a judiciously balanced approach in membrane design. Our proposal advocates for the use of radiation-induced RAFT polymerization as an efficient and innovative pathway for the design of well-defined advanced PEMs, particularly when aided by a suitable cross-linker. By harnessing the power of RAFT polymerization, our method minimizes sacrifices to membrane properties while simultaneously enhancing physicochemical properties through cross-linking, facilitated by the achieved homogeneity in the membrane structure.

## Supporting Information Available

Table showing the variation of degree of grafting as a function of irradiation dose and monomer concentration, core-level O 1s XPS spectra, surface elemental atomic compositions obtained by XPS, and AFM image of pristine ETFE film (PDF)

## References

[ref1] Johnsson F.; Kjärstad J.; Rootzén J. The Threat to Climate Change Mitigation Posed by the Abundance of Fossil Fuels. Climate Policy 2019, 19 (2), 258–274. 10.1080/14693062.2018.1483885.

[ref2] Chiari L.; Zecca A. Constraints of Fossil Fuels Depletion on Global Warming Projections. Energy Policy 2011, 39 (9), 5026–5034. 10.1016/j.enpol.2011.06.011.

[ref3] Barbir F. PEM Fuel Cells Theory and Practice, 1st ed.; Academic Press, 2005.

[ref4] Singla M. K.; Nijhawan P.; Oberoi A. S. Hydrogen Fuel and Fuel Cell Technology for Cleaner Future: A Review. Environmental Science and Pollution Research 2021, 28 (13), 15607–15626. 10.1007/s11356-020-12231-8. 33538968

[ref5] Zhang G.; Qu Z.; Wang N.; Wang Y. Spatial Distribution Characteristics in Segmented PEM Fuel Cells: Three-Dimensional Full-Morphology Simulation. Electrochim. Acta 2024, 484, 144061 10.1016/j.electacta.2024.144061.

[ref6] Winter M.; Brodd R. J. What Are Batteries, Fuel Cells, and Supercapacitors?. Chem. Rev. 2004, 104 (10), 4245–4270. 10.1021/cr020730k. 15669155

[ref7] Stoll J.; Zhao N.; Yuan X.-Z.; Girard F.; Kjeang E.; Shi Z. Impacts of Bubble Defects in Proton Exchange Membranes on Fuel Cell Performance and Durability. J. Power Sources 2024, 596, 234072 10.1016/j.jpowsour.2024.234072.

[ref8] Prykhodko Y.; Fatyeyeva K.; Hespel L.; Marais S. Progress in Hybrid Composite Nafion®-Based Membranes for Proton Exchange Fuel Cell Application. Chemical Engineering Journal 2021, 409, 127329 10.1016/j.cej.2020.127329.

[ref9] Ke Y.; Yuan W.; Zhou F.; Guo W.; Li J.; Zhuang Z.; Su X.; Lu B.; Zhao Y.; Tang Y.; Chen Y.; Song J. A Critical Review on Surface-Pattern Engineering of Nafion Membrane for Fuel Cell Applications. Renewable and Sustainable Energy Reviews 2021, 145, 110860 10.1016/j.rser.2021.110860.

[ref10] Nasef M. M. Radiation-Grafted Membranes for Polymer Electrolyte Fuel Cells: Current Trends and Future Directions. Chem. Rev. 2014, 114 (24), 12278–12329. 10.1021/cr4005499. 25418784

[ref11] Nasef M. M.; Hegazy E. S. A. Preparation and Applications of Ion Exchange Membranes by Radiation-Induced Graft Copolymerization of Polar Monomers onto Non-Polar Films. Progress in Polymer Science (Oxford) 2004, 29 (6), 499–561. 10.1016/j.progpolymsci.2004.01.003.

[ref12] Gubler L.; Gürsel S. A.; Scherer G. G. Radiation Grafted Membranes for Polymer Electrolyte Fuel Cells. Fuel Cells 2005, 5 (3), 317–335. 10.1002/fuce.200400078.

[ref13] Hao L. H.; Hieu D. T. T.; Luan L. Q.; Phuong H. T.; Dinh V.; Tuyen L. A.; Hong P. T. T.; Van Man T.; Tap T. D. Electron and Gamma Irradiation-induced Effects in Poly(Ethylene-co-tetrafluoroethylene) Films. J. Appl. Polym. Sci. 2022, 139 (29), e52620 10.1002/app.52620.

[ref14] Dargaville T. R.; George G. A.; Hill J. T.; Whittaker A. K. High Energy Radiation Grafting of Fluoropolymers. Prog. Polym. Sci. 2003, 28, 1355–1376. 10.1016/S0079-6700(03)00047-9.

[ref15] Aricò A. Investigation of Grafted ETFE-Based Polymer Membranes as Alternative Electrolyte for Direct Methanol Fuel Cells. J. Power Sources 2003, 123 (2), 107–115. 10.1016/S0378-7753(03)00528-7.

[ref16] Çelik G.; Barsbay M.; Güven O. Towards New Proton Exchange Membrane Materials with Enhanced Performance via RAFT Polymerization. Polym. Chem. 2016, 7 (3), 701–714. 10.1039/C5PY01527H.

[ref17] Grasselli M.; Betz N. Electron-Beam Induced RAFT-Graft Polymerization of Poly(Acrylic Acid) onto PVDF. Nucl. Instrum Methods Phys. Res. B 2005, 236 (1–4), 201–207. 10.1016/j.nimb.2005.04.026.

[ref18] Santos B. P. S.; Barbosa A. S.; Kodama Y.; de Queiroz T. B.; Santiago E. I. Tailoring Highly Stable Anion Exchange Membranes with Graft Molecular Structure Ordering Using Reversible Addition-Fragmentation Chain Transfer Polymerization for Alkaline Fuel Cells. J. Membr. Sci. 2023, 687, 122071 10.1016/j.memsci.2023.122071.

[ref19] Santos B. P. S.; Barbosa A. S.; Kodama Y.; de Queiroz T. B.; Santiago E. I. Tailoring Highly Stable Anion Exchange Membranes with Graft Molecular Structure Ordering Using Reversible Addition-Fragmentation Chain Transfer Polymerization for Alkaline Fuel Cells. J. Membr. Sci. 2023, 687, 122071 10.1016/j.memsci.2023.122071.

[ref20] Peng W.; Cai Y.; Fanslau L.; Vana P. Nanoengineering with RAFT Polymers: From Nanocomposite Design to Applications. Polym. Chem. 2021, 12 (43), 6198–6229. 10.1039/D1PY01172C.

[ref21] Zhao W.; Li C.; Chang J.; Zhou H.; Wang D.; Sun J.; Liu T.; Peng H.; Wang Q.; Li Y.; Whittaker A. K. Advances and Prospects of RAFT Polymerization-Derived Nanomaterials in MRI-Assisted Biomedical Applications. Prog. Polym. Sci. 2023, 146, 101739 10.1016/j.progpolymsci.2023.101739.

[ref22] Chapman R.; Jung K.; Boyer C. Photo RAFT Polymerization. In RAFT Polymerization; Wiley, 2021; pp 611–645. 10.1002/9783527821358.ch12.

[ref23] Zhu J.; Wang R.; Ma Z.; Zuo W.; Zhu M. Unleashing the Power of PET-RAFT Polymerization: Journey from Porphyrin-Based Photocatalysts to Combinatorial Technologies and Advanced Bioapplications. Biomacromolecules 2024, 25, 1371 10.1021/acs.biomac.3c01356. 38346318

[ref24] Ge Q.; Wang G.; Zhu X.; Yu W.; Zhou J.; Wu B.; Liu Y.; Zheng Z.; Yang Z.; Qian J. A Highly Stable Aliphatic Backbone from Visible Light-Induced RAFT Polymerization for Anion Exchange Membranes. Polym. Chem. 2021, 12 (39), 5574–5582. 10.1039/D1PY00867F.

[ref25] Deng C.; Webb M. A.; Bennington P.; Sharon D.; Nealey P. F.; Patel S. N.; de Pablo J. J. Role of Molecular Architecture on Ion Transport in Ethylene Oxide-Based Polymer Electrolytes. Macromolecules 2021, 54 (5), 2266–2276. 10.1021/acs.macromol.0c02424.

[ref26] Adamski M.; Peressin N.; Holdcroft S. On the Evolution of Sulfonated Polyphenylenes as Proton Exchange Membranes for Fuel Cells. Mater. Adv. 2021, 2 (15), 4966–5005. 10.1039/D1MA00511A.

[ref27] Ben youcef H.; Gubler L.; Yamaki T.; Sawada S.; Gürsel S. A.; Wokaun A.; Scherer G. G. Cross-Linker Effect in ETFE-Based Radiation-Grafted Proton-Conducting Membranes: II. Extended Fuel Cell Operation and Degradation Analysis. J. Electrochem. Soc. 2009, 156 (4), B532 10.1149/1.3082109.

[ref28] Gubler L.; Ben youcef H.; Gürsel S. A.; Wokaun A.; Scherer G. G. Cross-Linker Effect in ETFE-Based Radiation-Grafted Proton-Conducting Membranes: I. Properties and Fuel Cell Performance Characteristics. J. Electrochem. Soc. 2008, 155 (9), B921 10.1149/1.2951919.

[ref29] Kallio T.; Lundström M.; Sundholm G.; Walsby N.; Sundholm F. Electrochemical Characterization of Radiation-Grafted Ion-Exchange Membranes Based on Different Matrix Polymers. J. Appl. Electrochem. 2002, 32 (1), 11–18. 10.1023/A:1014222132075.

[ref30] Ben youcef H.; Gürsel S. A.; Wokaun A.; Scherer G. G. The Influence of Crosslinker on the Properties of Radiation-Grafted Films and Membranes Based on ETFE. J. Membr. Sci. 2008, 311 (1–2), 208–215. 10.1016/j.memsci.2007.12.015.

[ref31] Gubler L.; Prost N.; Gürsel S. A.; Scherer G. G. Proton Exchange Membranes Prepared by Radiation Grafting of Styrene/Divinylbenzene onto Poly(Ethylene-Alt-Tetrafluoroethylene) for Low Temperature Fuel Cells. Solid State Ion 2005, 176 (39–40), 2849–2860. 10.1016/j.ssi.2005.09.045.

[ref32] Gu R.; Xu W. Z.; Charpentier P. A. Synthesis of Graphene-Polystyrene Nanocomposites via RAFT Polymerization. Polymer (Guildf) 2014, 55 (21), 5322–5331. 10.1016/j.polymer.2014.08.064.

[ref33] Ben youcef H.; Gubler L.; Gürsel S. A.; Henkensmeier D.; Wokaun A.; Scherer G. G. Novel ETFE Based Radiation Grafted Poly(Styrene Sulfonic Acid-Co-Methacrylonitrile) Proton Conducting Membranes with Increased Stability. Electrochem commun 2009, 11 (5), 941–944. 10.1016/j.elecom.2009.02.047.

[ref34] Chapiro A. Radiation Chemisrty of Polymeric Systems; Wiley-Interscience: New York, 1962.

[ref35] Radice S.; Del Fanti N.; Castiglioni C.; Del Zoppo M.; Zerbi G. Vibrational Analysis as a Tool for Detecting Electronic Mobility. The Case of the Alternating Ethylene-Tetrafluoroethylene Copolymers. Macromolecules 1994, 27 (8), 2194–2199. 10.1021/ma00086a032.

[ref36] Nasef M. M.; Saidi H. Surface Studies of Radiation Grafted Sulfonic Acid Membranes: XPS and SEM Analysis. Appl. Surf. Sci. 2006, 252 (8), 3073–3084. 10.1016/j.apsusc.2005.05.013.

[ref37] Liang C. Y.; Krimm S. Infrared Spectra of High Polymers. VI. Polystyrene. J. Polym. Sci. 1958, 27 (115), 241–254. 10.1002/pol.1958.1202711520.

[ref38] Fan X.-D.; Bazuin C. G. Sulfonated Polystyrene Ionomers Neutralized by Bi- and Multifunctional Organic Cations. 1. An Infrared Spectroscopic Study. Macromolecules 1995, 28 (24), 8209–8215. 10.1021/ma00128a034.

[ref39] Yang J. C.; Jablonsky M. J.; Mays J. W. NMR and FT-IR Studies of Sulfonated Styrene-Based Homopolymers and Copolymers. Polymer (Guildf) 2002, 43 (19), 5125–5132. 10.1016/S0032-3861(02)00390-7.

[ref40] Yamamoto Y.; Higashi S.; Yamamoto K. XPS-depth Analysis Using C60 Ion Sputtering of Buried Interface in Plasma-treated Ethylene-tetrafluoroethylene-copolymer (ETFE) Film. Surf. Interface Anal. 2008, 40 (13), 1631–1634. 10.1002/sia.2884.

[ref41] Radice S.; Del Fanti N.; Castiglioni C.; Del Zoppo M.; Zerbi G. Vibrational Analysis as a Tool for Detecting Electronic Mobility. The Case of the Alternating Ethylene-Tetrafluoroethylene Copolymers. Macromolecules 1994, 27 (8), 2194–2199. 10.1021/ma00086a032.

[ref42] Xu X.; Kwok R. W. M.; Lau W. M. Surface Modification of Polystyrene by Low Energy Hydrogen Ion Beam. Thin Solid Films 2006, 514 (1–2), 182–187. 10.1016/j.tsf.2006.02.095.

[ref43] Alkan Gürsel S.; Gubler L.; Gupta B.; Scherer G. G. Radiation Grafted Membranes. Adv. Polym. Sci. 2008, 215 (1), 157–217. 10.1007/12_2008_153.

[ref44] Brack H.; Wyler M.; Peter G.; Scherer G. A Contact Angle Investigation of the Surface Properties of Selected Proton-Conducting Radiation-Grafted Membranes. Journal of Membrane Science - J. MEMBRANE SCI 2003, 214, 1–19. 10.1016/S0376-7388(02)00390-3.

[ref45] Gürsel S. A.; Schneider J.; Ben Youcef H.; Wokaun A.; Scherer G. G. Thermal Properties of Proton-conducting Radiation-grafted Membranes. J. Appl. Polym. Sci. 2008, 108 (6), 3577–3585. 10.1002/app.27947.

[ref46] Huang R. Y. M.; Shao P.; Burns C. M.; Feng X. Sulfonation of Poly(Ether Ether Ketone)(PEEK): Kinetic Study and Characterization. J. Appl. Polym. Sci. 2001, 82, 2651 10.1002/app.2118.

[ref47] Blanchard L. P.; Hornof V.; Lam H. H.; Malhotra S. L. Thermal Decomposition of Polystyrene, Polyoxyethylene Glycol and Their Mixtures by Thermogravimetric Techniques. Eur. Polym. J. 1974, 10 (11), 1057–1067. 10.1016/0014-3057(74)90071-8.

[ref48] Chen J.; Asano M.; Yamaki T.; Yoshida M. Preparation and Characterization of Chemically Stable Polymer Electrolyte Membranes by Radiation-Induced Graft Copolymerization of Four Monomers into ETFE Films. J. Membr. Sci. 2006, 269 (1–2), 194–204. 10.1016/j.memsci.2005.06.035.

[ref49] Li L.; Deng B.; Ji Y.; Yu Y.; Xie L.; Li J.; Lu X. A Novel Approach to Prepare Proton Exchange Membranes from Fluoropolymer Powder by Pre-Irradiation Induced Graft Polymerization. J. Membr. Sci. 2010, 346 (1), 113–120. 10.1016/j.memsci.2009.09.027.

[ref50] Zawodzinski T. A. Jr.; Springer T. E.; Uribe F.; Gottesfeld S. Characterization of Polymer Electrolytes for Fuel Cell Applications. Solid State Ion 1993, 60 (1–3), 199–211. 10.1016/0167-2738(93)90295-E.

[ref51] Kimura Y.; Asano M.; Chen J.; Maekawa Y.; Katakai R.; Yoshida M. Influence of Grafting Solvents on the Properties of Polymer Electrolyte Membranes Prepared by γ-Ray Preirradiation Method. Radiat. Phys. Chem. 2008, 77 (7), 864–870. 10.1016/j.radphyschem.2007.12.012.

[ref52] Hübner G.; Roduner E. EPR Investigation of HO/ Radical Initiated Degradation Reactions of Sulfonated Aromatics as Model Compounds for Fuel Cell Proton Conducting Membranes. J. Mater. Chem. 1999, 9 (2), 409–418. 10.1039/a807129b.

[ref53] Yu J.; Yi B.; Xing D.; Liu F.; Shao Z.; Fu Y.; Zhang H. Degradation Mechanism of Polystyrene Sulfonic Acid Membrane and Application of Its Composite Membranes in Fuel Cells. Phys. Chem. Chem. Phys. 2003, 5 (3), 611–615. 10.1039/b209020a.

[ref54] Nasef M. M.; Saidi H.; Yahaya A. H. Radiation-Induced Grafting of Styrene onto Polyethylene Films for Preparation of Cation Exchange Membranes: Effect of Crosslinking+. Journal of Applied Membrane Science & Technology 2017, 2 (1), na 10.11113/amst.v2i1.4.

[ref55] Borup R.; Meyers J.; Pivovar B.; Kim Y. S.; Mukundan R.; Garland N.; Myers D.; Wilson M.; Garzon F.; Wood D.; Zelenay P.; More K.; Stroh K.; Zawodzinski T.; Boncella J.; McGrath J. E.; Inaba M.; Miyatake K.; Hori M.; Ota K.; Ogumi Z.; Miyata S.; Nishikata A.; Siroma Z.; Uchimoto Y.; Yasuda K.; Kimijima K. I.; Iwashita N. Scientific Aspects of Polymer Electrolyte Fuel Cell Durability and Degradation. Chem. Rev. 2007, 107 (10), 3904–3951. 10.1021/cr050182l. 17850115

