# Supplementary material for: Designing Advanced Cross-Linked Proton Exchange Membranes with Enhanced Structural Homogeneity and Proton Conductivity via Radiation-Induced RAFT Polymerization
Source: ACS Omega. 2024 Jun 18;9(26):28194–206. doi: 10.1021/acsomega.4c01522 (PMC11223216; doi:10.1021/acsomega.4c01522)
Supplement: Supplementary file 1 — ao4c01522_si_001.pdf [file ao4c01522_si_001.pdf]

Supporting Information for

Designing Advanced Crosslinked Proton Exchange Membranes with Enhanced Structural Homogeneity and Proton Conductivity via Radiation-Induced RAFT Polymerization

Feyza Genç[a], Nazlıcan Yıldırım Kılıç[b], Murat Barsbay*[,a,b]

[a] *Polymer Chemistry Division, Department of Chemistry, Faculty of Science, Hacettepe University, 06800, Ankara, Turkey.*

[b] *Polymer Science and Technology Division, Institute of Science, Hacettepe University, 06800, Ankara, Turkey.*

Table S1. Radiation-induced grafting of styrene in the presence (entities 1-28) and absence (entity 29) of DVB. [St]/[DDMAT]=700, ETFE (0.01 g), solvent: toluene, dose rate: 1 kGy/h.

Entity No.	DVB, %	Rad. Dose, kGy	Monomer Conc.	DG, %
**1**	3.5	0.7	30	15
**2**	3.5	1.0	30	31
**3**	3.5	2.1	10	31
**4**	3.5	2.1	20	51
**5**	3.5	2.1	30	61
**6**	3.5	2.1	40	69
**7**	3.5	2.1	50	77
**8**	3.5	2.1	60	81
**9**	3.5	2.1	70	89
**10**	3.5	2.1	80	97
**11**	3.5	3.1	30	67
**12**	3.5	5.2	30	75
**13**	3.5	8.9	30	87
**14**	3.5	11.3	30	89
**15**	5	0.7	30	23
**16**	5	1.0	30	46
**17**	5	2.1	30	71
**18**	5	3.1	30	79
**19**	5	5.2	30	80
**20**	5	8.9	30	89
**21**	5	11.3	30	93
**22**	10	0.7	30	34
**23**	10	1.0	30	51
**24**	10	2.1	30	76
**25**	10	3.1	30	85
**26**	10	5.2	30	89
**27**	10	8.9	30	90
**28**	10	11.3	30	95
**29**	0	11.3	30	53

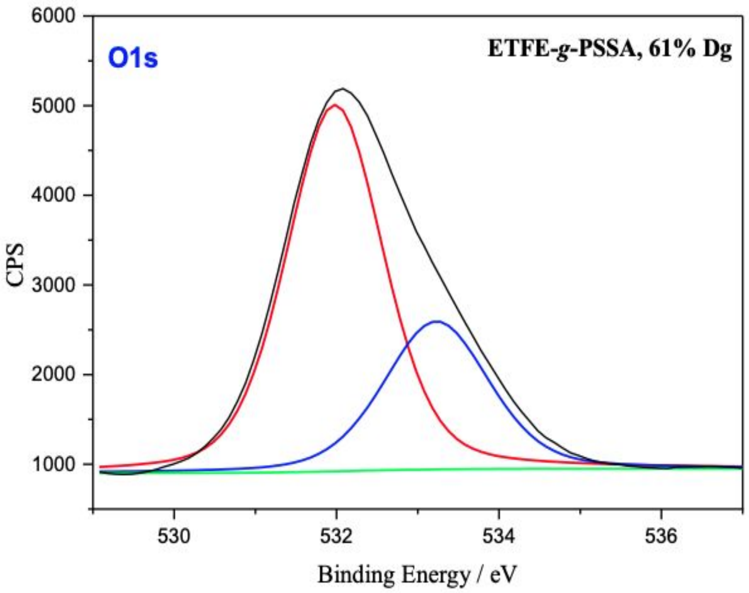

Figure S1. Core-level O1s XPS spectra of the sulfonated membrane (ETFE-*g*-PSSA) of the ETFE-*g*-PS (DG: 61%) film.

Table S2. Surface elemental atomic compositions obtained by XPS survey wide scans of pristine ETFE film, ETFE-*g*-PS films, and sulfonated membranes with different degrees of grafting.

Samples	F (%)	C (%)	O (%)	S (%)
**ETFE**	53.9	46.1	-	-
**% 39 ETFE-*g*-PS**	38.6	61.4	-	-
**% 61 ETFE-*g*-PS**	30.1	69.9	-	-
**% 87 ETFE-*g*-PS**	19.5	80.1	-	0.4
**% 39 ETFE-*g*-PSSA**	19.7	60.3	15.7	4.3
**% 61 ETFE-*g*-PSSA**	7.9	65.5	18.5	7.1
**% 87 ETFE-*g*-PSSA**	1.7	66.3	22.3	9.7

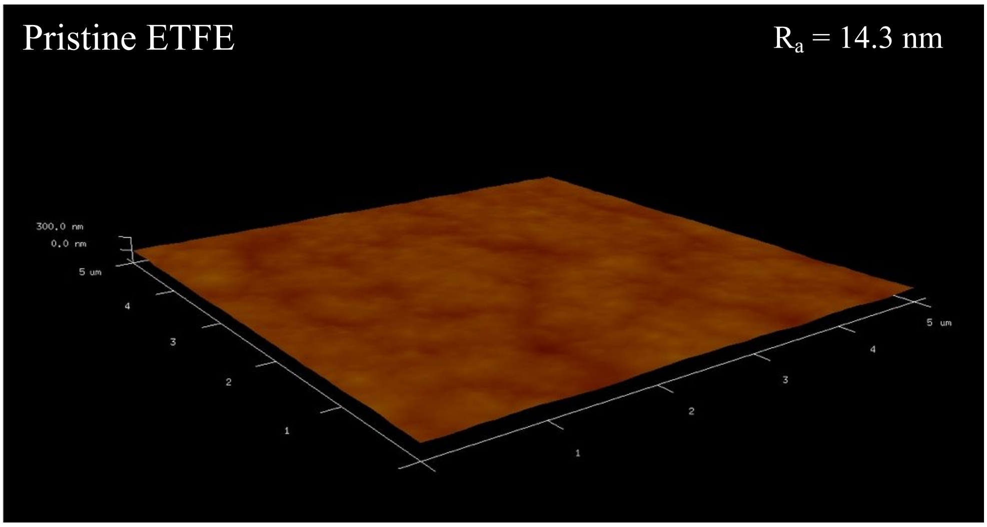

Figure S2. AFM image and roughness value (Ra) of pristine ETFE film.
